# Regularization in Retrieval-Driven Classification of Clustered Microcalcifications for Breast Cancer

**DOI:** 10.1155/2012/463408

**Published:** 2012-07-11

**Authors:** Hao Jing, Yongyi Yang, Robert M. Nishikawa

**Affiliations:** ^1^Department of Electrical and Computer Engineering, Illinois Institute of Technology, 3301 South Dearborn Street, Chicago, IL 60616, USA; ^2^Department of Radiology, The University of Chicago, 5841 S. Maryland Avenue, Chicago, IL 60637-1463, USA

## Abstract

We propose a regularization based approach for case-adaptive classification in computer-aided diagnosis (CAD) of breast cancer. The goal is to improve the classification accuracy on a query case by making use of a set of similar cases retrieved from an existing library of known cases. In the proposed approach, a prior is first derived from a traditional CAD classifier (which is typically pre-trained offline on a set of training cases). It is then used together with the retrieved similar cases to obtain an adaptive classifier on the query case. We consider two different forms for the regularization prior: one is fixed for all query cases and the other is allowed to vary with different query cases. In the experiments the proposed approach is demonstrated on a dataset of 1,006 clinical cases. The results show that it could achieve significant improvement in numerical efficiency compared with a previously proposed case adaptive approach (by about an order of magnitude) while maintaining similar (or better) improvement in classification accuracy; it could also adapt faster in performance with a small number of retrieved cases. Measured by the area of under the ROC curve (AUC), the regularization based approach achieved AUC = 0.8215, compared with AUC = 0.7329 for the baseline classifier (*P*-value = 0.001).

## 1. Introduction

**Figure 1:**
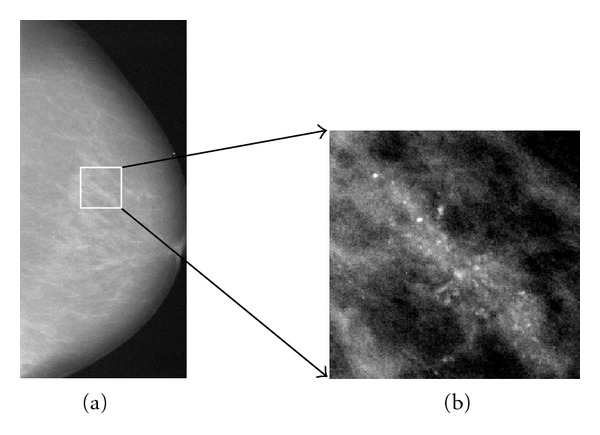
A mammogram image in CC view (a) and clustered microcalcifications in magnified view (b).

Clustered microcalcifications (MCs) can be an important early sign of breast cancer in women. MCs are calcium deposits of very small dimension and appear as granular bright spots in a mammogram (e.g., Figure 1). Due to their subtlety in appearance and variation in size and shape in mammogram images, accurate diagnosis of MC lesions as benign or malignant is a very challenging clinical problem for radiologists [1]. In recent years, there has been significant research in development of computer-aided diagnosis (CADx) techniques for clustered MCs, aiming to provide a second opinion to radiologists in their diagnosis in order to improve their performance and efficiency [1–3]. Laboratory observer studies have shown that with CADx radiologists can improve their biopsy recommendation by sending more cancer cases and fewer benign cases for biopsy [2–5].

In CADx, a pattern classifier is typically first pretrained on a set of existing cases and subsequently applied to predict the likelihood that a given lesion is malignant or benign. For this purpose, many different machine-learning methods have been investigated, for example, [6–9]. In recent years, content-based image retrieval (CBIR) has been studied as an alternative approach in CADx [10–12]. Instead of predicting likelihood, this approach is to provide radiologists with examples of lesions with known pathology that are similar to the lesion being evaluated. The purpose is to provide relevant information from the retrieved cases to boost the diagnostic accuracy on the case under consideration [13]. In the literature, there exist a number of studies on the predictive value of retrieved mammogram cases. For example, the correlation in disease condition between the query and retrieved cases was examined in [14, 15]. The fraction of malignant cases among all retrieved cases was used as a useful predictor for the query by Floyd et al. [16, 17]. The similarity level between a retrieved case and the query was used as a weighting factor in the prediction by Zheng et al. [18]. A genetic algorithm was used to adjust the weighting factors of the retrieved cases by Mazurowski et al. [19]. An observer study was used to investigate the potential diagnostic value of similar cases by Nakayama et al. [20].

Recently, we have been exploring a case-adaptive approach to boost the performance of a CADx classifier by using retrieved similar cases [21, 22]. The basic idea behind this approach is as follows: for a query case under consideration, we will first apply CBIR to obtain a set of similar cases from a reference library of known cases; we will then use these retrieved cases to modify the decision boundary of an existing classifier (baseline) in the neighborhood of the query case so as to improve its classification accuracy on the latter. In particular, we demonstrated this case-adaptive approach on a classifier based on logistic regression [22]. The adaptive classifier was obtained through retraining with a weighted mixture of the retrieved cases and the training cases of the baseline classifier. This adaptive approach was demonstrated to yield improved classification accuracy when compared to its baseline counterpart. Moreover, it could even outperform the classifier when it was retrained with all the cases in the reference library.

Based on this prior success, in this work, we further develop this case-adaptive classification approach by using a regularized adaptive classifier. One drawback of the adaptive classifier in [22] is the extra cost associated with retraining of the classifier for each query case. It also needs access to the training cases of the baseline classifier, which are required for retraining the classifier. To reduce this computational complexity associated with the adaptive classifier, we will use a prior to regularize the adaptive classifier as opposed to reusing the entire training set of the baseline classifier each time for a new query. This prior is derived from the baseline classifier, and it plays the following two key roles: (1) incorporate the information of the baseline classifier, and (2) prevent overfitting by the adaptive classifier when the number of retrieved samples is small. We will consider two specific forms for this prior: one is uniform for all query cases, and the other varies adaptively with the query. Our results demonstrate that such a regularized adaptive classifier not only can be much simpler computationally, it also can adapt faster in performance with a small number of retrieved cases.

Regularization techniques are often used in machine learning to deal with ill-posed problems or to prevent over-fitting by an underlying model. They usually assume the form of a penalty to the complexity of the model, such as *L*_2_-norm penalty in ridge regression [23], which penalizes the length of the solution in a least-square problem. In the well-known support vector machine (SVM) [24], the separation margin of the classifier is used in the form of an *L*_2_-norm penalty term. Parallel to *L*_2_-norm, *L*_1_-norm penalty has also been used for regularization, for example, the LASSO algorithm [25]. In this work, we will derive from the baseline classifier a regularization term for adaptive classification. The regularization term has the form of *L*_2_-norm penalty, which can also be viewed as a prior distribution of the solution.

The rest of the paper is organized as follows: The development of the adaptive classification schemes with regularization is given in Section 2. Details related to evaluation methods on CADx classification performance are described in Section 3. Experimental results and discussions are furnished in Section 4. Finally, conclusions are given in Section 5.

## 2. Regularized Adaptive Classification with ****Retrieval of Similar Cases

The problem we consider can be stated as follows: for a given query lesion **x**, we first obtain from a reference library a set of known cases which have similar image features to **x**; our goal is to make use of these similar, known cases to improve the classification accuracy on **x**. To motivate the proposed development, below we first briefly review the case-adaptive approach developed previously in [22]. For simplicity, our approach will be presented using a linear classifier. However, it can be readily extended to a nonlinear classifier by using the kernel trick as in [22].

### 2.1. Adaptive Classification Boosted with Similar Cases

Consider a linear classifier of the form:

(1) f(x)=wTx,

where **x** is a vector denoting an input pattern (i.e., lesion), and *f*(**x**) is the classifier output which is typically compared against an operating threshold for decision on **x**. For notational simplicity, in (1) the input vector **x** is augmented by a constant element 1 so that the bias term is absorbed into the discriminant vector **w**.

In practice, the unknown vector **w** is determined from a set of training samples {(**x**_*i*_, *y*_*i*_), *i* = 1,…, *N*}, where the labels *y*_*i*_ ∈ {0,1} are given for each sample **x**_*i*_. In [22], we considered logistic regression [26], in which **w** is determined by maximizing the following log-likelihood function:

(2) L(w)=∑i=1Nlog⁡⁡p(yi,xi;w),

where  *p*(*y*_*i*_ = 1, **x**_*i*_; **w**) = [1+exp⁡(−**w** ^*T*^ **x**_*i*_)]^−1^.

Now, consider a query lesion **x**, and a set of *N*_*r*_ retrieved cases {(**x**_*j*_^(*r*)^, *y*_*j*_^(*r*)^), *j* = 1,…, *N*_*r*_} which are similar to **x**. The adaptive classifier for **x** is obtained by modifying the objective function (2) as

(3) LAda(w)=∑i=1Nlog⁡⁡p(yi,xi;w)+∑j=1Nrβjlog⁡⁡p(yj(r),xj(r);w),

where the weighting factors *β*_*j*_ are defined according to the similarity of **x**_*j*_^(*r*)^ to the query **x **[22]. These factors are larger than 1 in magnitude. The idea is to put more emphasis on the retrieved samples, particularly those more similar to the query, so as to refine the decision boundary of the classifier in the neighborhood of **x**. For retrieval of similar cases, the Euclidean distance between their image features to the query was used in [22], and it is also used in this work.

### 2.2. Regularized Adaptive Classification with Uniform Prior

Observe that the objective function in (3) consists of two terms: the first term is that of the baseline classifier in (2), and the second term is the weighted sum of the log-likelihood of the retrieved cases. Conceptually, the first term can be viewed as a stabilizer for the adaptive classifier to avoid over-fitting for the retrieved cases (which would lead to poor generalization on the query). However, this term involves all the training cases of the baseline classifier, which can be computationally demanding particularly when the number of retrieved cases is much smaller than the number of existing training cases, that is, *N*_*r*_ ≪ *N*.

**Figure 2:**
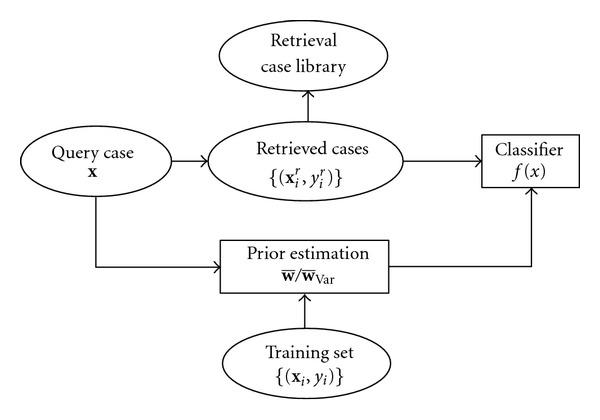
Diagram of retrieval-driven case-adaptive classification with regularization.

To address this problem, we propose a regularized approach for designing the adaptive classifier, as illustrated in Figure 2. The idea is to replace the baseline classifier term in (3) by a prior term on the discriminant vector **w**. Naturally, this prior term is desired to be predetermined from the training set {(**x**_*i*_, *y*_*i*_), *i* = 1,…, *N*}, so that the resulting adaptive classifier will be computationally more efficient for online implementation.

Let vector $\overset{-}{w}$ denote the solution of the baseline classifier in (2), that is, the likelihood function *L*(**w**) assumes maximum at $\overset{-}{w}$. Noting that the gradient ∇*L*(**w**) = 0 at $\overset{-}{w}$, we can apply Taylor series expansion about $\overset{-}{w}$ and rewrite *L*(**w**) as

(4) L(w)≈L(w−)+12(w−w−)T∇2L(w−)(w−w−).

Thus, we can rewrite the modified objective *L*_Ada_(**w**) in (3) as (after ignoring the constant term)

(5)   LAda(w)≈∑j=1Nrβjlog⁡⁡p(yj(r),xj(r);w)+12(w−w−)T∇2L(w−)(w−w−).

The second term in (5) can be viewed as a penalty term defined by a multivariate Gaussian prior which has mean $\overset{-}{w}$ and covariance matrix ${\left[-\nabla^{2}L\left(\overset{-}{w}\right)\right]}^{-1}$. Consequently, the objective function in (5) assumes the form of maximum *a posteriori* estimation (except that the log-likelihood terms of the retrieved cases are weighted according to their similarity level to the query).

Note that the Hessian matrix $\nabla^{2}L\left(\overset{-}{w}\right)$ in (5) can be pre-computed from the likelihood function *L*(**w**) of the trainings samples. By comparing to (3), we can see that the numerical complexity associated with the objective function in (5) is much reduced, because it consists of far fewer data terms than (3) when *N*_*r*_ ≪ *N*. Furthermore, there is no longer need in (5) to access the training cases, which can be advantageous in practice.

To further simplify the computational complexity of the adaptive classifier, in this study we assume that the components of **w** are independent and approximate the covariance matrix ${\left[-\nabla^{2}L\left(\overset{-}{w}\right)\right]}^{-1}$ in (5) by *C* ^−1^ **I**, where *C* is a constant. Upon such approximation, we can further simplify the objective function in (5) as

(6) LUni(w)=∑j=1Nrβjlog⁡⁡p(yj(r) ∣ xj(r);w)−C2||w−w−||2.

The constant *C* in (6) can be viewed as a parameter to control the influence of the regularization term, which has two important roles. First, it is used to prevent over-fitting by the adaptive classifier especially when *N*_*r*_ is small. Second, and more importantly, it is used to also enforce the fidelity of the adaptive classifier to the training cases {(**x**_*i*_, *y*_*i*_), *i* = 1,…, *N*} as in (3). Consequently, *L*_Uni_(**w**) in (6) consists of information from both the retrieved cases and the existing training cases. In particular, in the extreme case that *C* = 0, the objective function *L*_Uni_(**w**) in (6) simply amounts to retraining the classifier with only the retrieved cases; on the other hand, when *C* = *∞*, the adaptive classifier in (6) coincides with the baseline classifier $\overset{-}{w}$.

In this study, the weighting coefficient for a retrieved case **x**_*j*_^(*r*)^ in (6) is defined as

(7) βj=γj∑k=1Nrγk, where  γj=exp⁡(−||xj(r)−x||2σ2).

That is, *β*_*j*_ varies according to the distance between **x**_*j*_^(*r*)^ and **x**. The parameter *σ* is used to adjust the sensitivity of *β*_*j*_ with respect to the distance.

In our experiments, the Newton-Raphson algorithm was used for optimization of the adaptive classifier in (6). For completeness, the detailed algorithm is provided in the appendix

### 2.3. Adaptive Classification with Varying Regularization

In (6), the regularization term is the same for all query cases, the purpose of which is to keep the adaptive classifier from being too different from the baseline classifier. As an alternative, it might be advantageous to adjust this term according to the input feature of the query case. Below, we consider such an approach in which the mean vector $\overset{-}{w}$ in the regularization term is allowed to vary for each query case. That is, we modify the objective function in (6) as

(8) LVar⁡(w)=∑j=1Nrβjlog⁡⁡p(yj(r) ∣ xj(r);w)−C2||w−w−Var⁡||2,

where ${\overset{-}{w}}_{\mathrm{Var}}^{}$ is now varied with the query case **x**.

To determine the mean vector ${\overset{-}{w}}_{\mathrm{Var}}^{}$, we first pre-determine an adaptive vector **w**_*i*_ customized for each case in the training set, that is, {(**x**_*i*_, *y*_*i*_), *i* = 1,…, *N*}, as described below. Afterward, for a given query **x**, the mean vector ${\overset{-}{w}}_{\mathrm{Var}}^{}$ is interpolated from the adaptive vectors of the training cases according to their distances to the query. Specifically, we have

(9) w−Var⁡=∑i=1Nciwi,

where the weighting coefficients *c*_*i*_ are so defined that those cases closer to **x** will have more contributions to ${\overset{-}{w}}_{\mathrm{Var}}^{}$. In this study, the following is used for *c*_*i*_:

(10)   ci=αi∑k=1Nαk, where  αk=exp⁡(−||x−xi||2σ2).

To determine the adaptive vectors **w**_*i*_ for the cases in the training set {(**x**_*i*_, *y*_*i*_), *i* = 1,…, *N*}, we modify the objective function in (2) as

(11) L~(w1,…,wN)=∑i=1Nlog⁡⁡p(yi,xi;wi)−C′2∑i=1N||wi−w−i||2.

The rationale for the introduced penalty term in (11) is that those cases with similar features should also have similar discriminant vectors. The parameter *C*′ is used to control the trade-off between this penalty and the likelihood term. In (11), ${\overset{-}{w}}_{i}^{}$ denotes the weighted average of the adaptive vectors from the rest of the cases as in (9) with **x**_*i*_ treated as the query in (10).

## 3. Performance Evaluation

### 3.1. Dataset

In this study, we use a dataset as in our previous work [22]. This dataset consists of digitized, standard-view, screen-film mammographic images collected from two sources: one from the Department of Radiology, The University of Chicago (UC), and the other from the DDSM dataset maintained at The University of South Florida [27]. Altogether, there were a total of 1,006 cases (646 benign, 360 malignant) in the dataset, all containing clustered MCs. To characterize the MC lesions, we use a set of nine features previously determined in [22], namely, (a) number of MCs in the cluster, (b) density of the cluster, measured by the number of MCs in a unit area, (c) mean of the MC size in the cluster, (d) eccentricity of the cluster, (e) standard deviation of the distance from individual MCs to the geometric center of the cluster, (f) maximum of the mean intensity of MCs, (g) mean of the average intensity in each MC window, (h) standard deviation of the contrast of MCs, and (i) standard deviation of the 4th order central moment of MCs. These features are used to form a vector **x** for each lesion in the dataset. A detailed description for the construction of this dataset can be found in [22].

### 3.2. Experiment Setup

To demonstrate the proposed approach for case-adaptive classification, we used the following setting in our experiments. The dataset of all 1,006 cases was first randomly divided into three subsets, denoted by *S*_1_, *S*_2_, and *S*_3_, respectively, such that *S*_1_ and *S*_2_ consisted of 175 cases (100 benign, 75 malignant) each, and *S*_3_ had the remaining 656 cases (446 benign, 210 malignant). These three subsets were used as follows: *S*_1_ was used as the training set, *S*_2_ was used as the test set for performance evaluation, and *S*_3_ was set aside as a library of known cases for retrieval for adaptive classification. The distribution of the different cases among the three subsets was out of the consideration to balance the malignant and benign cases for both training and testing while maintaining a large number of cases for retrieval.

To avoid any potential bias, the training set *S*_1_ was used to determine the parameters *C*, *C*′ of the classifiers from the following candidate values: [0.001, 0.01, 0.05, 0.1, 0.2, 0.5, 1, 5, 10, 100] using a 10-fold cross-validation. For parameter *σ*, we follow our previous work [22] and set it to be 1.63, which corresponds to the 10th percentile of the inter-distance among training cases; the test set *S*_2_ was used exclusively for evaluation. When testing the adaptive classifiers, for each case in *S*_2_, a set of cases similar to the test case was retrieved from *S*_3_, and subsequently used to train the adaptive classifiers. The resulting classifiers were then applied to classify the test case. This was to ensure that the test case itself will not be used in any way for boosting the adaptive classifier.

To evaluate the classification performance, we conducted a receiver operating characteristic (ROC) analysis, which is now routinely used for performance evaluation in classification tasks. An ROC curve is a plot of the classification sensitivity (i.e., true positive fraction) as the ordinate versus the specificity (i.e., false positive fraction) as the abscissa; for a given classifier, it is obtained by continuously varying the threshold associated with its decision function. As a summary measure of overall diagnostic performance, the area under an ROC curve (denoted by AUC) is used. A larger AUC means better classification performance. In our experiments the ROCKIT program [28] was used to calculate the AUC values for the different classifiers.

To remove the effect of case distributions, we applied a bootstrapping methodology for testing the performance of the classifiers. A total of 2,000 bootstrap sample sets were used [29, 30], of which each was obtained by sampling with replacements from the cases in *S*_2_. The classifier performance was subsequently obtained over each bootstrap sample set.

In our evaluation, the proposed adaptive approach was compared against the following different classifiers: (1) the baseline classifier (2) trained with *S*_1_; (2) the adaptive classifier in (3). In addition, to demonstrate the effect of regularization, we also tested the adaptive classifier without using regularization, that is, by ignoring the regularization term in (6). Finally, we also considered the classifier trained with both *S*_1_ and *S*_3_, which represents the scenario where all the cases in the retrieval library were used for training the classifier.

## 4. Results and Discussions

### 4.1. Regularized Adaptive Classification

**Figure 3:**
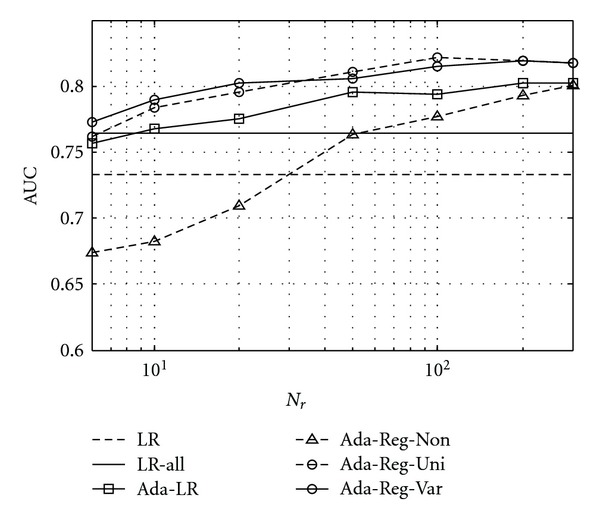
Classification performance (AUC) achieved by the case-adaptive classifiers with/without regularization (Ada-Reg-Non, Ada-Reg-Uni and Ada-Reg-Var). The number of retrieved cases *N*_*r*_ was varied from 6 to 300. For comparison, results are also shown for the baseline classifier (LR), the classifier trained with all the available cases (LR-all), and the adaptive classifier in [22] (Ada-LR).

In Figure 3, we show the performance results obtained by the regularized adaptive classifiers with uniform prior in (6) (Ada-Reg-Uni) and with adaptive prior in (8) (Ada-Reg-Var). To demonstrate the effect of retrieved cases, the results are shown for the number of retrieved cases *N*_*r*_ varied from 6 to 300. For comparison, results are also given in Figure 3 for the following classifiers: the baseline classifier (LR), the adaptive classifier previously developed in [22] (Ada-LR), the adaptive classifier without regularization (Ada-Reg-Non), and, finally, the classifier trained with *all* the cases in *S*_1_ and *S*_3_ (LR-all).

From Figure 3, it can be seen that the regularized adaptive classifiers Ada-Reg-Uni and Ada-Reg-Var both could outperform the baseline classifier LR. In particular, with *N*_*r*_ = 50, Ada-Reg-Uniachieved AUC = 0.8111 and Ada-Reg-Var achived AUC = 0.8059, compared with AUC = 0.7329 for the baseline classifier LR (*P*-value = 0.001 for Ada-Reg-Uni, and 0.004 for Ada-Reg-Var). With *N*_*r*_ = 100, Ada-Reg-Uni obtained its best performance of AUC = 0.8215; Ada-Reg-Var achieved its best performance of AUC = 0.8192 with *N*_*r*_ = 200. However, no further improvement was observed when *N*_*r*_ was increased beyond 200. We believe that this is because that the benefit from additional retrieved cases diminishes as they are not sufficiently similar to the query.

Furthermore, from Figure 3, it can be seen that when *N*_*r*_ < 50 Ada-Reg-Var and Ada-Reg-Uni are both higher in AUC than Ada-LR. This indicates that Ada-Reg-Var and Ada-Reg-Uni could adapt faster to the local decision boundary with a small number of retrieved cases. This could be attributed to the use of the prior in the regularized adaptive classifiers. With *N*_*r*_ further increased (above 100), Ada-Reg-Uni and Ada-Reg-Var became similar in performance; this is because with a large *N*_*r*_ the retrieved cases became more influential than the prior on the classifier.

The respective effects of retrieved cases and regularization can be illuminated by examining the results achieved by Ada-Reg-Non, that is, when no regularization was used in the adaptive classifier. With *N*_*r*_ < 50, Ada-Reg-Non was much lower in performance than even the baseline classifier LR; this was clearly due to the issue of over-fitting. However, with increased *N*_*r*_, its performance AUC was improved from 0.7633 with *N*_*r*_ = 50 to 0.7927 with *N*_*r*_ = 200, approaching its regularized counterparts.

Furthermore, the regularized adaptive classifiers Ada-Reg-Uni and Ada-Reg-Var could also outperform the baseline classifier LR-all (AUC = 0.7643) which was trained with *all* the available cases in *S*_1_ and *S*_3_. Specifically, the Ada-Reg-Uni and Ada-Reg-Var outperformed LR-all with *P*-value = 0.004 (*N*_*r*_ = 100) and *P*-value = 0.005 (*N*_*r*_ = 200), respectively.

### 4.2. Effect of Regularization

**Figure 4:**
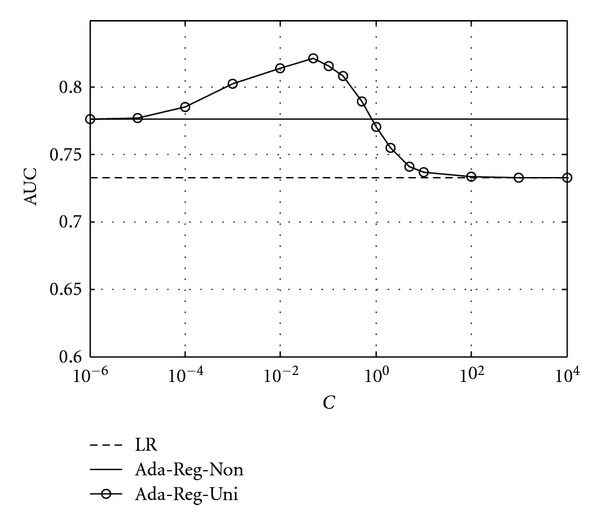
Effect of regularization prior in adaptive classifier Ada-Reg-Uni with parameter *C* varied between two extreme cases (i.e., *C* → 0 for no regularization, and *C* → *∞* regularization alone) and fixed *N*_*r*_ = 100.

The rationale behind the proposed regularization-based approach for adaptive classification is to use a prior to regularize the adaptive classifier in order to prevent it from over-fitting to the retrieved cases. As can be seen from (6), the regularization parameter *C* is used to control the balance between the retrieved cases and the baseline classifier. A larger *C* means more influence of the prior on the adaptive classifier (and less influence by the retrieved cases), and vice versa. To demonstrate this effect, in Figure 4, we show the resulting performance achieved by the classifier Ada-Reg-Uni with the parameter *C* varied over a large range. The number of retrieved cases *N*_*r*_ was fixed at 100. Note that, as *C* → 0, the classifier performance approaches that of Ada-Reg-Non (i.e., retrieval only, *N*_*r*_ = 100); on the other hand, as *C* → *∞*, the classifier performance approaches that of the baseline classifier LR (i.e., no retrieval). The best performance was obtained with *C* varied between these two extreme cases.

### 4.3. Execution Time

**Figure 5:**
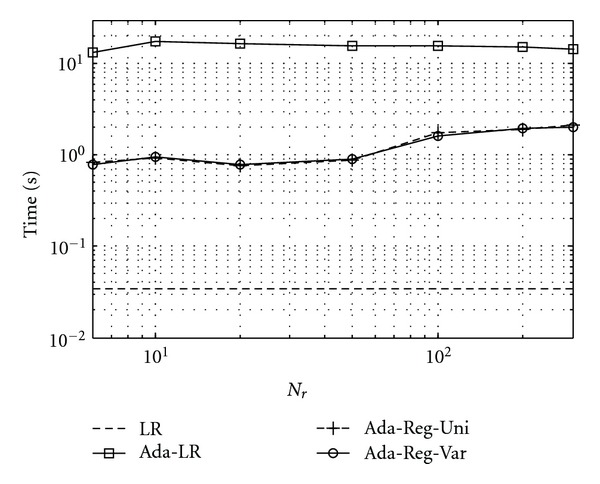
Execution time for classifying all test cases in *S*_2_ by the different classifiers. The regularization approaches (Ada-Reg-Uni and Ada-Reg-Var) are similar in execution time, and are notably faster than the adaptive classifier in [22] (Ada-LR).

In Figure 5, we show the execution time taken for classifying the cases in the test set by the different adaptive classifiers Ada-Reg-Uni, Ada-Reg-Var, and Ada-LR. For comparison, results are also shown for the baseline classifier LR. Our implementation was in MATLAB on a 2-GHz PC. As can be seen, the regularized classifiers Ada-Reg-Uni and Ada-Reg-Var were similar in execution time. While slower than the baseline classifier LR, both were notably faster than Ada-LR. Specifically, with *N*_*r*_ < 100, the regularization-based approaches were about 10 times faster. For *N*_*r*_ larger than 100, the execution time increased for the adaptive classifiers as more samples were used in training, but still much lower than that of Ada-LR. Interestingly, the execution time for Ada-LR slightly decreased with *N*_*r*_ larger than 10. We believe that this was due to improved conditioning in the Hessian matrix of the objective function with increased *N*_*r*_. The higher numerical efficiency of the regularized classifiers over the adaptive classifier was due to their much simplified objective functions in which only retrieved cases were used.

## 5. Conclusion

In this work, we investigated a regularization based approach for case-adaptive classification of microcalcification lesions in mammograms. Deviating from a previously developed adaptive approach, in which a set of retrieved cases was used in conjunction with the training cases of a baseline classifier to re-retrain an adaptive classifier, we derived a prior in place of the baseline classifier as a regularization term in the adaptive classifier. This prior was used together with the retrieved cases from a reference library to optimize the classification on a query case. Our goal was to reduce the numerical complexity associated with online training of the adaptive classifier. We explored two different forms for the regularization prior: one is invariant for the different query cases and the other is allowed to vary with respect to the features of the query cases. We demonstrated the proposed regularization approach on a dataset of 1,006 cases. The results show that it could achieve significant improvement in numerical efficiency (around 10 times in execution speed) while maintaining similar (or better) improvement in classification accuracy compared to a previous nonregularization approach. The regularization approach was also observed to achieve faster adaption in performance with a small number of retrieved cases.

## Appendix

### A. Optimization in Regularized Adaptive Classifier

We used the Newton-Raphson method to solve the optimization problems associated with the regularized adaptive classifiers. In particular, consider the objective function in (6), which we rewrite as:

(A.1) L(w)=∑i=1Nβilog⁡p(yi ∣ xi;w)−C2||w−w−||2,

where *p*(*y*_*i*_, **x**_*i*_; **w**) is defined as

(A.2) p(yi=1,xi;w)=11+exp⁡(−wTxi),p(yi=0,xi;w)=1−p(yi=1,xi;w).

Substituting (A.2) into (A.1), we get

(A.3) L(w)=∑i=1Nγi{yiwTxi−log⁡(1+exp⁡(wTxi))}−C2||w−w−||2.

The gradient and Hessian matrix of *L*(**w**) can then be obtained as

(A.4) ∇L(w)=XTΓ(y−p)−C(w−w−),∇2L(w)=−XTΓWX−CI,

where **y** is the column vector of sample labels *y*_*i*_, **X** is the input matrix, **p** is the column vector of probabilities *p*(*y*_*i*_, **x**_*i*_; **w**), Γ, and **W** are diagonal matrices with Γ_*i*,*i*_ = *γ*_*i*_, **W**_*i*,*i*_ = *p*(*y*_*i*_, **x**_*i*_; **w**)(1 − *p*(*y*_*i*_, **x**_*i*_; **w**)), respectively.

The Newton-Raphson update is computed iteratively as

(A.5) wnew=wold−(∇2L(wold))−1∇L(wold)=wold+(XTΓWoldX+CI)−1×[XTΓ(y−p)−C(wold−w−)].
